# Measurement properties of instruments used to measure health-related quality of life in pediatric and adults patients with inherited epidermolysis bullosa: A systematic review and meta-analysis protocol

**DOI:** 10.1371/journal.pone.0332844

**Published:** 2025-09-19

**Authors:** Mario Gómez-Martínez, Greta Arias-Merino, Juan Benito-Lozano, Ana Villaverde-Hueso, Renata Linertová, Verónica Alonso-Ferreira

**Affiliations:** 1 Institute of Rare Diseases Research (IIER), Instituto de Salud Carlos III (ISCIII), Madrid, Spain; 2 Centre for Biomedical Network Research on Rare Diseases (CIBERER), Instituto de Salud Carlos III, Madrid, Spain; 3 Canary Islands Health Research Institute Foundation (FIISC), Santa Cruz de Tenerife, Spain; 4 Research Network on Chronicity, Primary Care, and Health Promotion (RICAPPS), Instituto de Salud Carlos III, Madrid, Spain; 5 Spanish Network of Agencies for Health Technology Assessment for the National Health Service (RedETS), Spain; Freelance Medical Research and Writing, UNITED KINGDOM OF GREAT BRITAIN AND NORTHERN IRELAND

## Abstract

Inherited Epidermolysis Bullosa (EB) is a group of rare, genetic skin diseases characterized by extreme fragility of the skin and mucous membranes, leading to blistering and wounds in response to minimal trauma or friction. These clinical manifestations significantly reduce health-related quality of life (HRQoL). The objective of this protocol article is to provide information about the methods planned to be used to assess the measurement properties of HRQoL instruments specifically developed for EB patients of all age groups through a systematic review and meta-analysis. The protocol followed the Preferred Reporting Items for Systematic Reviews and Meta-analyses Protocols (PRISMA-P) guideline. The literature search will be conducted in PubMed, Web of Science (WOS) and EMBASE, including terminology that aligns with the four key elements of the COSMIN (COnsensus-based Standards for the selection of health Measurement INstruments) research question (construct, target population, measurement properties and type of PROM), as well as the terminology proposed by COSMIN for measurement properties. Studies that include information on measurement properties (specifically, validity and/or reliability) with a sample of patients with inherited EB will be selected. Both title and abstract screening and full text review, will be conducted by two independent reviewers using the Rayyan tool. In addition, the risk of bias will be assessed using the COSMIN-Risk of Bias checklist. The data from each study and each measurement property will be summarized in accordance with the COSMIN guidelines. The evidence gathered will strive to adjudicate data on measurements properties of HRQoL instruments used in EB patients, and the limitations of the future systematic review will be discussed. Ultimately, results of the future systematic review will help develop more personalized guidelines for the assessment of HRQoL in EB patients of all age groups. The protocol is registered in OSF with registration number vrm87: https://osf.io/vrm87/

## Introduction

Inherited Epidermolysis Bullosa (EB) is a group of autosomal dominant or autosomal recessive rare disorders for which there is currently no cure [1–3]. Based on the European Union (EU) definition of a Rare Disease (RD) established in 1999 (present in fewer than 5 individuals per 10,000 people) [4], EB is a RD as it affects from 1 to 9 individuals per 100,000 inhabitants in Europe and between 1 in 85,000 to 1 in 500,000 people worldwide [3]. Four major types of EB have been identified: simplex EB (EBS), junctional EB (JEB), dystrophic EB (DEB), and Kindler EB [1,2]. DEB is caused by *COL7A1* [5–7] mutations and KS by *FERMT1* [8]. In contrast, EBS and JEB are associated with different genes, primarily the following: EBS with *KRT5, KRT14, PLEC, KLHL24, DST, EXPH5*, and *CD151*; and JEB with *LAMA3, LAMB3, LAMC2, COL17A1, ITGA6, ITGB4*, and *ITGA3* [1,2,9].

Clinical manifestations (including skin contracture and other systemic complications such as digestive and oropharyngeal manifestations) typically appear at birth or within the first few months of life and persist throughout life [1,10–12]. These complications may lead to many consequences (e.g., nutritional deficiencies and resultant anemia) and affect variably the quality of life across pediatric and adult populations including walking and daily living activities [13–16]. Survival and functional prognosis in EB are highly variable and depends on both the subtype and associated comorbidities [3,12,14,17–21]. The diagnosis of this RD often requires skin biopsy and genetic testing to determine the specific EB subtype [9,17,22,23]. EB treatment is palliative in nature, focusing on preventing the formation of skin lesions by eliminating sources of friction or trauma and managing associated pain and pruritus [23,24].

To date, various generic and disease-specific instruments have been used to assess HRQoL in pediatric and adult patients with EB, but there is no consensus about the most appropriate tool to be used in either group [16,25–28]. Various EB-specific instruments have been used to assess HRQoL in EB patients of both adult and pediatric groups. However, drawing on their literature review, the European Academy of Dermatology and Venereology (EADV) recommends that the Instrument for Quality of Life in Epidermolysis Bullosa (IntoDermQoL-EB, developed in 2019), be used in children under 5 years of age [29]; the Instrument for Scoring Clinical Outcomes of Research for Epidermolysis Bullosa (Iscor-EB-p) be used in children aged 5 to 10 years [30]; and that the Quality of Life in Epidermolysis Bullosa (QoLEB) and/or the Iscor-EB be used in individuals over 10 years of age [29–33].

Up to now, no systematic review (SR) has assessed the methodological quality of the studies, analyzed the measurement properties of EB-specific HRQoL questionnaires, or examined validated age-adapted versions of EB-specific instruments to determine their reliability. Accordingly, the aim of this research protocol framed within the project Changes in the Socio-economic Burden of Epidermolysis Bullosa in Europe (BUR-EB), is to outline the methodology for a SR and meta-analysis aimed at identifying, assessing and synthesizing the evidence on the measurement properties (reliability and validity) of HRQoL instruments specifically designed for patients with inherited EB. This objective stems from a research question (What is the reliability and validity of the instruments currently used to measure HRQoL in EB pediatric and adult patients?) developed in the context of BUR-EB. This project aims to estimate the socio-economic burden of EB in seven EU countries (Spain, France, Austria, Germany, Italy, Hungary, and Bulgaria) and compare the findings with data collected ten years earlier in the BURQOL-RD project, which focused on EB and other RD. Ultimately, BUR-EB intends to provide evidence that supports future investigations into novel therapeutic approaches, healthcare service design, and the development of public policies addressing the EB [34].

## Materials and methods

### Followed guidelines

The protocol is reported according to the Preferred Reporting Items for Systematic Review and Meta-analysis Protocols (PRISMA-P) 2015 checklist [35] (S1 Checklist). The proposed SR of HRQoL instruments in patients with inherited EB will be conducted following the Joanna Briggs Institute (JBI) criteria for systematic reviews of measurement properties in 2019 [36] and the PRISMA-COSMIN (COnsensus-based Standards for the selection of health Measurement INstruments) checklist for systematic reviews of outcome measurement instruments [37] (S2 Checklist). The properties to be assessed will be validity and reliability, as defined in the COSMIN taxonomy [38,39]. The protocol for this SR is registered at OSF (https://osf.io/vrm87/).

### Eligibility criteria

The publications included in this SR will meet the following inclusion criteria:

(1) They must address the measurement properties of the HRQoL instruments specific for inherited EB, including at least the following properties: reliability (test-retest, inter-rater, intra-rater and/or internal consistency) and/or validity (content, criterion and/or construct) as defined in the COSMIN taxonomy [38,39].
(2) The study sample must include patients with inherited EB of any subtype (EBS, DEB, JEB, and Kindler EB).
(3) A HRQoL instrument specific to inherited EB must have been administered.
(4) Quantitative studies of any design may be eligible for inclusion, including both observational and interventional types.
(5) Studies may include different age groups, with no restrictions on geographical area, context, ethnicity or sex.
(6) Studies published in any language will be included without restrictions.

The following studies will be excluded:

(1) Systematic reviews, meta-analysis, and other types of literature reviews.
(2) Studies that use HRQoL questionnaires specific to inherited EB but do not report measurement properties, or that use them solely as a reference for validating another instrument.
(3) Articles reporting overlapping results (salami-sliced) that provide less information.
(4) Articles not retrieved after contacting the authors.

### Information sources

Systematic searches will be conducted in MEDLINE (through PubMed), Web of Science (WOS) (trough Clarivate), and EMBASE (trough Elsevier) databases. Initially, PsycINFO (through EBSCO) was also considered; however, its exclusion was based on a preliminary analysis that did not retrieve any publications mentioning both inherited EB and HRQoL. The search will be conducted without restrictions on date or language.

### Preliminary search strategy

The search terms will refer to the measurement properties of instruments used to measure the HRQoL of individuals affected with inherited EB of any age. For the measurement properties search filter part, COSMIN-recommended terms developed in 2009 [40], which show high sensitivity for PubMed/MEDLINE, WOS and EMBASE, will be used [41]. Minor adjustments were made to the PubMed measurement properties filter to ensure the search was conducted correctly. The criteria from the Peer Review of Electronic Search Strategies (PRESS) guideline for SR were considered in order to optimize the search strategy [42–45]. The peer review was conducted by experts (GAM and MGM) within the research group specializing in the subject area.

Essential elements of the research question were included, and the COSMIN exclusion filters for psychometric properties were removed from the search strategies applied in PubMed and WOS. These decisions were intended to prevent excessive search limitations and the exclusion of pertinent references. The preliminary search strategy in each database can be found in S3 Appendix.

In addition, the search terms were based on the four key elements of the COSMIN research question [38]:

- Target population: patients with inherited EB, including all synonyms for this RD.
- Type of Patient-Reported Outcome Measure (PROM): all HRQoL specific instruments identified for patients with inherited EB were added (Iscor EB-p; QoLEB and InToDermQoL-EB).
- Construct: terms related to HRQoL were incorporated.
- Measurement properties: terms related to measurement properties compiled in a COSMIN search filter were included, for PubMed, WOS and EMBASE [41].

### Selection process

After the search, two reviewers will independently review the publications by title and abstract using the Rayyan tool, developed in 2016 [46]. They will then perform the full-text review. If it is not possible to access the full text of a publication, the authors will be contacted to provide the information to avoid bias. If the full text is in a language unfamiliar to the reviewers, a specialized translation service will be employed. In cases of disagreement, discrepancies will be resolved by a third reviewer.

The phases of the SR study selection process will be presented in the PRISMA flow diagram [47]. The number of articles identified, included, and excluded, as well as the reasons for exclusions, will be described in detail.

### Data collection process

For each eligible publication, data will be independently extracted by two reviewers. Disagreements will be resolved through discussion and, if necessary, a third reviewer will be consulted. Data will be extracted into S4 Table.

### Data items

The following information will be extracted from the included studies. 1) study characteristics (study, objectives, sample, life stages, EB subtype, setting, geographical area). 2) characteristics of the EB-specific HRQoL instruments (name, mode of administration, dimensions, items). 3) measurement properties: validity (content, criterion and/or construct), reliability (test-retest, inter-rater, intra-rater and/or internal consistency) and others (e.g., responsiveness or measurement error) (see S4 Table).

### Risk of bias assessment in individual studies

The methodological quality of the studies and of the HRQoL instruments specific to inherited EB will be assessed using the COSMIN risk of bias checklist [48]. This checklist consists of ten boxes related to measurement properties and other relevant characteristics that include different elements that can be rated from “Very good” to “Inadequate”. Ratings are assigned according to the ‘worst score counts’ principle, whereby the lowest item score determines the overall rating of the study. Specifically, the following aspects will be evaluated: the development of PROMs, content validity, structural validity, internal consistency, cross-cultural validity, reliability, measurement error, criterion validity, hypothesis testing for construct validity, and responsiveness. The assessment will be carried out independently by two reviewers and, in case of disagreement, a third reviewer will be consulted.

### Measurement properties

The information on measurement properties from each study will be rated according to the COSMIN criteria for good measurement properties, using three possible ratings: sufficient (+), insufficient (–), or indeterminate (?) [48]. In general, a sufficient (+) rating indicates that there is evidence that the specific measurement property of the PROM is of good quality. An insufficient (–) rating indicates that the property is of poor quality. An indeterminate (?) rating means that something was done, but the available information is insufficient to rate the results [38]. This task will be conducted independently by two reviewers. Discrepancies between their assessments will be resolved through discussion to reach consensus. In cases where consensus cannot be reached, a third reviewer will adjudicate.

### Synthesis methods

The data collected from each study selected for each measurement property of the EB-specific HRQoL instruments will be synthesized according to the COSMIN criteria for good measurement properties: and rated as sufficient (+), insufficient (–) or indeterminate (?) [48]. When all results are rated as either sufficient or insufficient, they may be synthesized qualitatively or quantitatively through statistical analysis. In cases of inconsistency, various strategies will be employed to summarize the evidence.

The meta-analysis of the previously described psychometric indicators will be performed if the selected studies’ characteristics permit and they provide the necessary data. It will be assumed that statistical heterogeneity is present and that the mean of the effect sizes is not consistent across studies; therefore, a random-effects model will be used. To assess heterogeneity, Cochrane’s Q test and the I² statistic will be employed. As a general guide, heterogeneity of 0–40% is usually unimportant; whereas 30–60% may indicate moderate, 50–90% substantial, and 75–100% considerable heterogeneity [49,50]. Additionally, where appropriate, funnel plots will be provided to identify publication bias and the effect size of studies with small sample size, as well as sensitivity analyses. Depending on the data obtained, meta-regression and/or subgroup analyses may be performed [49,51,52].

The results will be presented through tabulated summaries and/or comprehensive graphs for the reader, accompanied by a descriptive summary according to the PRISMA 2020 statement [53]. In the case of the meta-analysis, the results will be presented using forest plots.

### Certainty assessment

The level of evidence will be assessed using the GRADE (Grading of Recommendations Assessment, Development, and Evaluation) approach, as recommended by COSMIN [38].The level of evidence will be rated as high”, “moderate”, “low” or “very low” [48]. If a result is rated as indeterminate, grading the quality of evidence will not be required.

The assessment of the level of evidence will be conducted independently by two reviewers. In cases of disagreement, efforts will be made to reach a consensus. If consensus cannot be achieved a third reviewer will be consulted to resolve the discrepancy.

### Preliminary timeframe

The SR has not yet started, and the results are expected in the first half of 2026. In the following lines a timeline for each stage is presented, including the publication selection, data extraction, risk of bias assessment, and data synthesis. The SR is expected to be completed within 7–8 months. The tasks and their corresponding approximate timeframes are outlined below, structured by quarter:

- Fourth quarter of 2025. Comprehensive search in PubMed, EMBASE and WOS with adjustments in search strategy if necessary (2–3 weeks); Selection process (screening titles, abstracts, and full texts) (2–3 weeks); Data extraction (2–3 weeks); Risk of bias assessment in individual studies (4–6 weeks); Measurement properties assessment (2–3 weeks).
- First quarter of 2026. Data synthesis methods (narrative synthesis and meta-analysis) (3–4 weeks); Level of evidence assessment (GRADE) (3–4 weeks); Drafting of the first version of the manuscript (1–4 weeks).
- Second quarter of 2026. Review of the manuscript by coauthors and changes (6–8 weeks); Final changes and manuscript submission (3–4 weeks).
- Third quarter of 2026. Editorial process (peer-review and acceptance) (10–12 weeks).
- Fourth quarter of 2026. Conference presentations (abstract submission) and Online dissemination (social media, repositories) (10–12 weeks).

### Protocol deviation

Amendments to the protocol will be made if necessary and may include the following: changes to the inclusion and/or exclusion criteria, adjustments to the search strategy and modifications to the data extraction methods. The implementation of the meta-analysis may also not be feasible, at least for some HRQoL instruments, and even if the meta-analysis is feasible, some statistical analyses (e.g., subgroup analyses and publication bias assessment) included in a meta-analysis may not be feasible [49,54]. Any updates to the protocol will be documented on the OSF website.

### Data sharing

The following information will be included in a future publication reporting the results of the SR and meta-analysis described in this protocol, either as tables within the manuscript or as supplementary material. This will cover information from each publication, in accordance with S4 Table. It will present the evaluation of each measurement property in each selected study. A summary of the evidence will be provided by psychometric property and by type of EB-specific instrument. Finally, the quality of the identified evidence will be evaluated.

The objective is to ensure transparency and reproducibility of the process, in accordance with the principles of open science.

### Patient and public involvement

There was no involvement of patients or the public in the design, execution, reporting, or dissemination of this research protocol.

## Discussion

For the assessment of HRQoL in patients with inherited EB, generic, dermatological-specific, and disease-specific instruments have been used. However, evaluations using generic instruments in patients with inherited EB have raised issues with content validity and ceiling effect. These problems are more frequently observed in individuals with lower HRQoL, such as those with recessive DEB, as already reported in 2009 and 2024. Moreover, non-specific questionnaires do not capture the full complexity of the manifestations and symptoms of EB, and consequently, their impact on HRQoL [27,55].

Regarding EB-specific instruments, there is no synthesized evidence, any SR or meta-analysis on their measurement properties in the publications reporting these properties. Narrative synthesis and meta-analysis will provide information on the measurement properties of EB-specific HRQoL instruments, enabling the development of evidence-based recommendations for patients with EB worldwide. Furthermore, no SRs have assessed which instruments are of sufficient quality to be administered to different age groups. By including both pediatric and adult populations, it will be possible to compare measurement instruments between these two patient groups.

The adoption of both the JBI criteria for systematic reviews of measurement properties [36] and the PRISMA-COSMIN methodology for systematic reviews of OMIs [37,38] will enhance the quality and transparency of the SR by providing an evaluation of the evidence found. Furthermore, there is a clear set of inclusion and exclusion criteria and a detailed description of the publication selection process which will prevent arbitrary decision-making. Moreover, to minimize selection bias as much as possible – such as that which could arise from excluding, among others, letters to the editor or publications in languages other than English the only types of publications excluded are SR, meta-analyses, and other forms of literature reviews. In addition, if the full text of any publication is not available, efforts will be made to retrieve it via the Carlos III Health Institute Library and the National Council for Science, Technology and Innovation (Concytec) Library. If retrieval through these means is unsuccessful, the corresponding authors will be contacted twice within a 15-day interval between the first and second attempt. Similarly, efforts have been made to minimize misclassification bias by specifically defining the type of population responding to the HRQoL questionnaires (patients with hereditary EB) as stated in the inclusion criteria.

Among the potential limitations, there is a concern regarding representativeness of different parts of the world because RD awareness, diagnosis, and report are limited across the globe, particularly in low and middle-income countries. Moreover, there is a possible risk of full-text bias if it is not feasible to access all publications included in the full-text review phase. Likewise, it is possible that future articles included will have a limited sample of patients since it is an RD. Furthermore, there may be a limited number of publications, which could influence the strength of the evidence found. This could be attributed to the exclusion of generic and dermatology-related questionnaires. Additionally, challenges may arise in data synthesis and meta-analysis due to potential heterogeneity among the studies. Similarly, the studies may fail to report all required measurement properties or may do so inadequately or unclearly. However, by defining precise inclusion and exclusion criteria, this protocol ensures a clear approach to study selection, screening and data extraction. Moreover, there may be a lack of validation of the instruments across different linguistic or cultural contexts.

Transparency in the methods and processes used is upheld by adhering to PRISMA-P guidelines.

### Ethics and dissemination of the results

The BUR-EB project, to which this protocol belongs, was approved by the Ethics Committee (CEIm) of the University Hospital Complex of the Canary Islands (26/01/2023). Due to the specific characteristics of this protocol’s design, additional ethical committee approval was not required. The results of the SR will be submitted for publication to a specialized open-access, peer-reviewed scientific journal. They will also be presented at various conferences and seminars within the relevant field. During these scientific events, information regarding the most suitable instruments for the assessment of HRQoL in EB patients, as identified in the SR, will be provided.

## Conclusion

The results that will be found are expected to be beneficial to more precisely quantify the impact that EB has on patients´ HRQoL. Furthermore, the recommendations resulting from this work will be valuable for researchers in future studies, healthcare professionals in their evaluations, and those involved in the creation and implementation of public policies related to EB.

## Supporting information

S1 Checklist PRISMA-P (Preferred Reporting Items for Systematic review and Meta-Analysis Protocols) 2015 checklist. (DOCX)

S2 Checklist PRISMA-COSMIN checklist for Outcome Measurement Instruments (OMIs). (DOCX)

S3 Appendix Preliminary search strategy. (DOCX)

S4 Table Data extraction sheet. (DOCX)
