# Supplementary material for: Measurement properties of instruments used to measure health-related quality of life in pediatric and adults patients with inherited epidermolysis bullosa: A systematic review and meta-analysis protocol
Source: PLoS One. 2025 Sep 19;20(9):e0332844. doi: 10.1371/journal.pone.0332844 (PMC12448333; doi:10.1371/journal.pone.0332844)
Supplement: S2 Checklist — (DOCX) [file pone.0332844.s002.docx]

**S2 Checklist. PRISMA-COSMIN checklist for Outcome Measurement Instruments (OMIs)**

Article: Measurement properties of instruments used to measure health-related quality of life in pediatric and adults patients with inherited epidermolysis bullosa: a systematic review and meta-analysis protocol

| **Section and Topic** | **#** | **Checklist item^a^** | **Location** |
| --- | --- | --- | --- |
| TITLE | | | |
| Title | 1 | Identify the report as a systematic review and include as applicable the following (in any order): outcome domain of interest, population  of interest, name/type of OMIs of interest, and measurement properties of interest. | Page 1 |
| ABSTRACT | | | |
| OPEN SCIENCE | | | |
| Funding^b^ | 2.2 | Specify the primary source of funding for the review. | Submission form |
| Registration | 2.3 | Provide the register name and registration number. | Page 2 |
| BACKGROUND | | | |
| Objectives | 2.4 | Provide an explicit statement of the main objective(s) or question(s) the review addresses. | Page 2 |
| METHODS | | | |
| Eligibility criteria | 2.5 | Specify the inclusion and exclusion criteria for the review. | Page 2 |
| Information sources | 2.6 | Specify the information sources (e.g., databases, registers) used to identify studies and the date when each was last searched. | Page 2 |
| Risk of bias | 2.7 | Specify the methods used to assess risk of bias in the included studies. | Page 2 |
| Measurement properties | 2.8 | Specify the methods used to rate the results of a measurement property. | Page 2 |
| Synthesis methods | 2.9 | Specify the methods used to present and synthesize results. | Page 2 |
| RESULTS | | | |
| Included studies | 2.10 | Give the total number of included OMIs and study reports. | N/A |
| Synthesis of results | 2.11 | Present the syntheses of results of OMIs, indicating the certainty of the evidence. | N/A |
| DISCUSSION | | | |
| Limitations of evidence | 2.12 | Provide a brief summary of the limitations of the evidence included in the review (e.g., study risk of bias, inconsistency, and imprecision). | N/A |
| Interpretation | 2.13 | Provide a general interpretation of the results and important implications. | N/A |
| PLAIN LANGUAGE SUMMARY | | | |
| Plain language summary | 3 | If allowed by the journal, provide a plain language summary with background information and key findings. | N/A |
| OPEN SCIENCE | | | |
| Registration and protocol | 4a | Provide registration information for the review, including register name and registration number, or state that the review was not registered. | Pages 2,5 |
|  | 4b | Indicate where the review protocol can be accessed, or state that a protocol was not prepared. | Pages 2,5 |
|  | 4c | Describe and explain any amendments to information provided at registration or in the protocol. | N/A |
| Support | 5 | Describe sources of financial or non-financial support for the review, and the role of the funders in the review. | Submission form |
| Competing interests | 6 | Declare any competing interests of review authors. | Submission form |
| Availability of data, code, and other materials | 7 | Report which of the following are publicly available and where they can be found: template data collection forms; data extracted from  included studies; data used for all analyses; analytic code; any other materials used in the review. | Page 8-10, S3 |
| INTRODUCTION | | | |
| Rationale | 8 | Describe the rationale for the review in the context of existing knowledge. | Pages 3-4 |
| Objectives | 9 | Provide an explicit statement of the objective(s) or question(s) the review addresses and include as applicable the following (in any  order): outcome domain of interest, population of interest, name/type of OMIs of interest, and measurement properties of interest. | Page 4 |
| METHODS | | | |
| Followed guidelines | 10 | Specify, with references, the methodology and/or guidelines used to conduct the systematic review. | Pages 5 |
| Eligibility criteria | 11 | Specify the inclusion and exclusion criteria for the review. | Pages 5-6 |
| Information sources | 12 | Specify all databases, registers, preprint servers, websites, organizations, reference lists and other sources searched or consulted to  identify studies. Specify the date when each source was last searched or consulted. | Page 6 |
| Search strategy | 13 | Present the full search strategies for all databases, registers, and websites, including any filters and limits used. | Page 6-7, S3 |
| Selection process | 14 | Specify the methods used to decide whether a study met the inclusion criteria of the review, e.g., including how many reviewers  screened each record and each report retrieved, whether they worked independently, and if applicable, details of automation tools/AI  used in the process. | Page 7-8 |
| Data collection process | 15 | Specify the methods used to collect data from reports, e.g., including how many reviewers collected data from each report, whether  they worked independently, any processes for obtaining or confirming data from study investigators, and if applicable, details of  automation tools/AI used in the process. | Page 8 |
| Data items | 16 | List and define which data were extracted (e.g., characteristics of study populations and OMIs, measurement properties’ results, and  aspects of feasibility and interpretability). Describe methods used to deal with any missing or unclear information. | Page 8 |
| Study risk of bias assessment | 17 | Specify the methods used to assess risk of bias in the included studies, e.g., including details of the tool(s) used, how many reviewers  assessed each study and whether they worked independently, and if applicable, details of automation tools/AI used in the process. | Pages 8-9 |
| Measurement properties | 18 | Specify the methods used to rate the results of a measurement property for each individual study and for the summarized or pooled  results, e.g., including how many reviewers rated each study and whether they worked independently. | Pages 9 |
| Synthesis methods | 19a | Describe the processes used to decide which studies were eligible for each synthesis. | Page 9-10 |
|  | 19b | Describe any methods used to synthesize results. | Page 9-10 |
|  | 19c | If applicable, describe any methods used to explore possible causes of inconsistency among study results (e.g., subgroup analysis). | N/A |
|  | 19d | If applicable, describe any sensitivity analyses conducted to assess robustness of the synthesized results. | N/A |
| Certainty assessment | 20 | Describe any methods used to assess certainty (or confidence) in the body of evidence. | Page 10 |
| Formulating recommendations | 21 | If appropriate, describe any methods used to formulate recommendations regarding the suitability of OMIs for a particular use. | N/A |
| RESULTS | | | |
| Study selection | 22a | Describe the results of the search and selection process, from the number of records identified in the search to the number of study  reports included in the review, ideally using a flow diagram. If applicable, also report the final number of OMIs included and the number  of study reports relevant to each OMI. [T] | N/A |
|  | 22b | Cite study reports that might appear to meet the inclusion criteria, but which were excluded, and explain why they were excluded. | N/A |
|  |  |  |  |
| OMI characteristics | 23a | Present characteristics of each included OMI, with appropriate references. [T] | N/A |
|  | 23b | If applicable, present interpretability aspects for each included OMI. [T] | N/A |
|  | 23c | If applicable, present feasibility aspects for each included OMI. [T] | N/A |
| Study characteristics | 24 | Cite each included study report evaluating one or more measurement properties and present its characteristics. [T] | N/A |
| Risk of bias in studies | 25 | Present assessments of risk of bias for each included study. [T] | N/A |
| Results of individual studies | 26 | For all measurement properties, present for each study: (a) the reported result and (b) the rating against quality criteria, ideally using  structured tables or plots. [T] | N/A |
| Results of syntheses | 27a | Present results of all syntheses conducted. For each measurement property of an OMI, present: (a) the summarized or pooled result and  (b) the overall rating against quality criteria. [T] | N/A |
|  | 27b | If applicable, present results of all investigations of possible causes of inconsistency among study results. | N/A |
|  | 27c | If applicable, present results of all sensitivity analyses conducted to assess the robustness of the synthesized results. | N/A |
| Certainty of evidence | 28 | Present assessments of certainty (or confidence) in the body of evidence for each measurement property of an OMI assessed. [T] | N/A |
| Recommendations | 29 | If appropriate, make recommendations for suitable OMIs for a particular use. | N/A |
| DISCUSSION | | | |
| Discussion | 30a | Provide a general interpretation of the results in the context of other evidence. | Pages 12-14 |
|  | 30b | Discuss any limitations of the evidence included in the review. | Pages 12-14 |
|  | 30c | Discuss any limitations of the review processes used. | Pages 12-14 |
|  | 30d | Discuss implications of the results for practice, policy, and future research. | Pages 12-14 |

It is strongly recommended that this checklist is used in conjunction with the PRISMA-COSMIN for OMIs 2024 Explanation and Elaboration (E&E) document for important clarification on the checklist items. The PRISMA-COSMIN for OMIs 2024 statement checklist is distributed under the terms of the Creative Commons license.

^a^ If an item is marked with [T], a template for data visualization is available. These templates can be downloaded from [www.prisma-cosmin.ca](http://www.prisma-cosmin.ca) ^b^ Item #2.1 in the PRISMA-COSMIN for OMIs 2024 Abstracts checklist refers to the title. Item #2.1 in the Abstracts checklist is identical to item #1 in the Full Report checklist.

*From:* Elsman EBM, Mokkink LB, Terwee CB, Beaton D, Gagnier JJ, Tricco AC, et al. Guideline for reporting systematic reviews of outcome measurement instruments (OMIs): PRISMA-COSMIN for OMIs 2024. *Quality of Life Research* (2024), doi: <https://doi.org/10.1007/s11136-024-03634-y>.

N/A: Not Applicable
