# Supplementary material for: Measurement properties of instruments used to measure health-related quality of life in pediatric and adults patients with inherited epidermolysis bullosa: A systematic review and meta-analysis protocol
Source: PLoS One. 2025 Sep 19;20(9):e0332844. doi: 10.1371/journal.pone.0332844 (PMC12448333; doi:10.1371/journal.pone.0332844)
Supplement: S3 Appendix — (DOCX) [file pone.0332844.s003.docx]

**S3 Appendix. preliminary search strategy**

**PubMed**

Strategy for using the filter

| Search terms | Query |
| --- | --- |
| Construct | ((("Quality of Life"[Mesh] OR "Quality of life"[Title/Abstract] OR "Health Related Quality of life" [Title/Abstract] OR "QOL" [Title/Abstract] OR "HRQOL" [Title/Abstract] ))) |
|  | AND |
| Target population | (("Epidermolysis Bullosa"[Mesh] OR "Epidermolysis Bullosa Simplex"[Mesh] OR "Epidermolysis Bullosa, Junctional"[Mesh] OR "Epidermolysis Bullosa Dystrophica"[Mesh] OR "kindler-syndrome" OR "Poikiloderma of Kindler" [Supplementary Concept] OR "Inherited epidermolysis bullosa" OR (("Congenital Abnormalities"[Majr]) AND ("Skin Manifestations"[Mesh])))) |
|  | AND |
| Type of PROM | (("Infants and Toddlers Dermatology Quality of Life" OR "InToDermQoL" OR "Instrument for Scoring Clinical Outcomes of Research for Epidermolysis Bullosa" OR "iscorEB-p" OR "iscorEB" OR "Quality of Life in Epidermolysis Bullosa" OR "QOLEB")) |
|  | AND |
| Measurement properties | (((instrumentation[sh] OR methods[sh] OR "Validation Study" [Publication Type] OR "Comparative Study"[pt] OR "psychometrics"[MeSH] OR psychometr*[tiab] OR clinimetr*[tw] OR clinometr*[tw] "Outcome Assessment, Health Care"[Mesh] OR "outcome assessment"[tiab] OR "outcome measure*"[tw] OR "observer variation"[MeSH] OR "observer variation"[tiab] OR "Health Status Indicators"[Mesh] OR "reproducibility of results"[MeSH] OR reproducib*[tiab] OR "discriminant analysis"[MeSH] OR reliab*[tiab] OR unreliab*[tiab] OR valid*[tiab] OR "coefficient of variation"[tiab] OR coefficient[tiab] OR homogeneity[tiab] OR homogeneous[tiab] OR "internal consistency"[tiab] OR (cronbach*[tiab] AND (alpha[tiab] OR alphas[tiab])) OR (item[tiab] AND (correlation*[tiab] OR selection*[tiab] OR reduction*[tiab])) OR agreement[tw] OR precision[tw] OR imprecision[tw] OR "precise values"[tw] OR test-retest[tiab] OR (test[tiab] AND retest[tiab]) OR (reliab*[tiab] AND (test[tiab] OR retest[tiab])) OR stability[tiab] OR interrater[tiab] OR inter-rater[tiab] OR intrarater[tiab] OR intra-rater[tiab] OR intertester[tiab] OR inter-tester[tiab] OR intratester[tiab] OR intra-tester[tiab] OR interobserver[tiab] OR inter-observer[tiab] OR intraobserver[tiab] OR intra-observer[tiab] OR intertechnician[tiab] OR inter-technician[tiab] OR intratechnician[tiab] OR intra-technician[tiab] OR interexaminer[tiab] OR inter-examiner[tiab] OR intraexaminer[tiab] OR intra-examiner[tiab] OR interassay[tiab] OR inter-assay[tiab] OR intraassay[tiab] OR intra-assay[tiab] OR interindividual[tiab] OR inter-individual[tiab] OR intraindividual[tiab] OR intra-individual[tiab] OR interparticipant[tiab] OR inter-participant[tiab] OR intraparticipant[tiab] OR intra-participant[tiab] OR kappa[tiab] OR kappa’s[tiab] OR kappas[tiab] OR repeatab*[tw] OR ((replicab*[tw] OR repeated[tw]) AND (measure[tw] OR measures[tw] OR findings[tw] OR result[tw] OR results[tw] OR test[tw] OR tests[tw])) OR generaliza*[tiab] OR generalisa*[tiab] OR concordance[tiab] OR (intraclass[tiab] AND correlation*[tiab]) OR discriminative[tiab] OR "known group"[tiab] OR "factor analysis"[tiab] OR "factor analyses"[tiab] OR "factor structure"[tiab] OR "factor structures"[tiab] OR dimension*[tiab] OR subscale*[tiab] OR (multitrait[tiab] AND scaling[tiab] AND (analysis[tiab] OR analyses[tiab])) OR "item discriminant"[tiab] OR "interscale correlation*"[tiab] OR error[tiab] OR errors[tiab] OR "individual variability"[tiab] OR "interval variability"[tiab] OR "rate variability"[tiab] OR (variability[tiab] AND (analysis[tiab] OR values[tiab])) OR (uncertainty[tiab] AND (measurement[tiab] OR measuring[tiab])) OR "standard error of measurement"[tiab] OR sensitiv*[tiab] OR responsive*[tiab] OR (limit[tiab] AND detection[tiab]) OR "minimal detectable concentration"[tiab] OR interpretab*[tiab] OR ((minimal[tiab] OR minimally[tiab] OR clinical[tiab] OR clinically[tiab]) AND (important[tiab] OR significant[tiab] OR detectable[tiab]) AND (change[tiab] OR difference[tiab])) OR (small*[tiab] AND (real[tiab] OR detectable[tiab]) AND (change[tiab] OR difference[tiab])) OR "meaningful change"[tiab] OR "ceiling effect"[tiab] OR "floor effect"[tiab] OR "Item response model"[tiab] OR IRT[tiab] OR Rasch[tiab] OR "Differential item functioning"[tiab] OR DIF[tiab] OR "computer adaptive testing"[tiab] OR "item bank"[tiab] OR "cross-cultural equivalence"[tiab]))) |

**EMBASE**

Strategy for using the filter

| Search terms | Query |
| --- | --- |
| Construct | ((('quality of life'/exp OR 'quality of life':ti,ab,kw OR 'health related quality of life':ti,ab,kw OR 'qol':ti,ab,kw OR 'hrqol':ti,ab,kw))) |
|  | AND |
| Target population | (('epidermolysis bullosa'/exp OR 'epidermolysis bullosa simplex'/exp OR 'epidermolysis bullosa'/exp OR 'epidermolysis bullosa dystrophica'/exp OR 'kindler-syndrome' OR 'poikiloderma of kindler' OR 'inherited epidermolysis bullosa' OR (('congenital disorder'/exp/mj) AND ('skin manifestation'/exp)))) |
|  | AND |
| Type of PROM | (('infants and toddlers dermatology quality of life' OR 'intodermqol' OR 'instrument for scoring clinical outcomes of research for epidermolysis bullosa' OR 'iscoreb-p' OR 'iscoreb' OR 'quality of life in epidermolysis bullosa' OR 'qoleb')) |
|  | AND |
| Measurement properties | 'intermethod comparison'/exp OR 'data collection method'/exp OR 'validation study'/exp OR 'feasibility study'/exp OR 'pilot study'/exp OR 'psychometry'/exp OR 'reproducibility'/exp OR reproducib*:ab,ti OR 'audit':ab,ti OR psychometr*:ab,ti OR clinimetr*:ab,ti OR clinometr*:ab,ti OR 'observer variation'/exp OR 'observer variation':ab,ti OR 'discriminant analysis'/exp OR 'validity'/exp OR reliab*:ab,ti OR valid*:ab,ti OR 'coefficient':ab,ti OR 'internal consistency':ab,ti OR (cronbach*:ab,ti AND ('alpha':ab,ti OR 'alphas':ab,ti)) OR 'item correlation':ab,ti OR 'item correlations':ab,ti OR 'item selection':ab,ti OR 'item selections':ab,ti OR 'item reduction':ab,ti OR 'item reductions':ab,ti OR 'agreement':ab,ti OR 'precision':ab,ti OR 'imprecision':ab,ti OR 'precise values':ab,ti OR 'test-retest':ab,ti OR ('test':ab,ti AND 'retest':ab,ti) OR (reliab*:ab,ti AND ('test':ab,ti OR 'retest':ab,ti)) OR 'stability':ab,ti OR 'interrater':ab,ti OR 'inter-rater':ab,ti OR 'intrarater':ab,ti OR 'intra-rater':ab,ti OR 'intertester':ab,ti OR 'inter-tester':ab,ti OR 'intratester':ab,ti OR 'interobeserver':ab,ti OR 'inter-observer':ab,ti OR 'intraobserver':ab,ti OR 'intertechnician':ab,ti OR 'inter-technician':ab,ti OR 'intratechnician':ab,ti OR 'interexaminer':ab,ti OR 'inter-examiner':ab,ti OR 'intraexaminer':ab,ti OR 'interassay':ab,ti OR 'inter-assay':ab,ti OR 'intraassay':ab,ti OR 'intra-assay':ab,ti OR 'interindividual':ab,ti OR 'inter-individual':ab,ti OR 'intraindividual':ab,ti OR 'intra-individual':ab,ti OR 'interparticipant':ab,ti OR 'inter-participant':ab,ti OR 'intraparticipant':ab,ti OR 'kappa':ab,ti OR 'kappas':ab,ti OR 'coefficient of variation':ab,ti OR repeatab*:ab,ti OR ((replicab*:ab,ti OR 'repeated':ab,ti) AND ('measure':ab,ti OR 'measures':ab,ti OR 'findings':ab,ti OR 'result':ab,ti OR 'results':ab,ti OR 'test':ab,ti OR 'tests':ab,ti)) OR generaliza*:ab,ti OR generalisa*:ab,ti OR 'concordance':ab,ti OR ('intraclass':ab,ti AND correlation*:ab,ti) OR 'discriminative':ab,ti OR 'known group':ab,ti OR 'factor analysis':ab,ti OR 'factor analyses':ab,ti OR 'factor structure':ab,ti OR 'factor structures':ab,ti OR 'dimensionality':ab,ti OR subscale*:ab,ti OR 'multitrait scaling analysis':ab,ti OR 'multitrait scaling analyses':ab,ti OR 'item discriminant':ab,ti OR 'interscale correlation':ab,ti OR 'interscale correlations':ab,ti OR (('error':ab,ti OR 'errors':ab,ti) AND (measure*:ab,ti OR correlat*:ab,ti OR evaluat*:ab,ti OR 'accuracy':ab,ti OR 'accurate':ab,ti OR 'precision':ab,ti OR 'mean':ab,ti)) OR 'individual variability':ab,ti OR 'interval variability':ab,ti OR 'rate variability':ab,ti OR 'variability analysis':ab,ti OR ('uncertainty':ab,ti AND ('measurement':ab,ti OR 'measuring':ab,ti)) OR 'standard error of measurement':ab,ti OR sensitiv*:ab,ti OR responsive*:ab,ti OR ('limit':ab,ti AND 'detection':ab,ti) OR 'minimal detectable concentration':ab,ti OR interpretab*:ab,ti OR (small*:ab,ti AND ('real':ab,ti OR 'detectable':ab,ti) AND ('change':ab,ti OR 'difference':ab,ti)) OR 'meaningful change':ab,ti OR 'minimal important change':ab,ti OR 'minimal important difference':ab,ti OR 'minimally important change':ab,ti OR 'minimally important difference':ab,ti OR 'minimal detectable change':ab,ti OR 'minimal detectable difference':ab,ti OR 'minimally detectable change':ab,ti OR 'minimally detectable difference':ab,ti OR 'minimal real change':ab,ti OR 'minimal real difference':ab,ti OR 'minimally real change':ab,ti OR 'minimally real difference':ab,ti OR 'ceiling effect':ab,ti OR 'floor effect':ab,ti OR 'item response model':ab,ti OR 'irt':ab,ti OR 'rasch':ab,ti OR 'differential item functioning':ab,ti OR 'dif':ab,ti OR 'computer adaptive testing':ab,ti OR 'item bank':ab,ti OR 'cross-cultural equivalence':ab,ti |

**Web of Science (WOS)**

Strategy for using the filter

| Search terms | Query |
| --- | --- |
| Construct | TS=(“Quality of Life” OR ”Health Related Quality of life” OR “HRQOL” OR “QOL”) |
|  | AND |
| Target population | ((TI=(epidermolysis bullosa OR epidermolysis bullosa simplex OR Junctional Epidermolysis Bullosa OR Dystrophic Epidermolysis Bullosa OR kindler-syndrome ) ) OR AB=(epidermolysis bullosa OR epidermolysis bullosa simplex OR Junctional Epidermolysis Bullosa OR Dystrophic Epidermolysis Bullosa OR kindler-syndrome )) OR AB=("Congenital Disorders" AND skin AND manifestations ) OR AB=("Skin Disorders" AND "Congenital Disorders") |
|  | **AND** |
| Type of PROM | TS=("Infants and Toddlers Dermatology Quality of Life" OR “InToDermQoL” OR “Instrument for Scoring Clinical Outcomes of Research for Epidermolysis Bullosa” OR "iscorEB" OR "iscorEB-p" OR “Quality of Life in Epidermolysis Bullosa” OR "QOLEB") |
|  | **AND** |
| Measurement properties | TS=(instrumentation OR methods) OR TS=(“validation study” OR “comparative study”) OR TS=(Psychometrics) OR (TI=(psychometr*)) OR AB=(psychometr*) OR TS=(clinimetr* OR clinometr*) OR TS=(“Outcome Assessment, Health Care”) OR (TI=(“outcome assessment”)) OR AB=(“outcome assessment”) OR TS=(“outcome measure*”) OR TS=(“Observer Variation”) OR TI=(“observer variation”) OR AB=(“observer variation”) OR TS=(“Health Status Indicators”) OR TS=(“Reproducibility of Results”) OR (TI=(reproducib*)) OR AB=(reproducib*) OR TS=(“Discriminant Analysis”) OR (TI=((reliab* OR unreliab* OR valid* OR “coefficient of variation” OR coefficient OR homogeneity OR homogeneous OR “internal consistency”))) OR AB=((reliab* OR unreliab* OR valid* OR “coefficient of variation” OR coefficient OR homogeneity OR homogeneous OR “internal consistency”)) OR (TI=(( cronbach* AND (alpha OR alphas)))) OR AB=(( cronbach* AND (alpha OR alphas))) OR (TI=((item AND (correlation* OR selection* OR reduction*)))) OR AB=((item AND (correlation* OR selection* OR reduction*))) OR TS=(agreement) OR TS=(precision) OR TS=(imprecision) OR TS=(“precise values”) OR (TI=(test-retest)) OR AB=(test-retest) OR (TI=((test AND retest))) OR AB=((test AND retest)) OR (TI=((reliab* AND (test OR retest)))) OR AB=((reliab* AND (test OR retest))) OR (TI=(stability)) OR AB=(stability) OR (TI=((interrater OR inter-rater OR intrarater OR intra-rater))) OR AB=((interrater OR inter-rater OR intrarater OR intra-rater)) OR (TI=((intertester OR inter-tester OR intratester OR intra-tester))) OR AB=((intertester OR inter-tester OR intratester OR intra-tester)) OR (TI=((interobserver OR inter-observer OR intraobserver OR intra-observer))) OR AB=((interobserver OR inter-observer OR intraobserver OR intra-observer)) OR (TI=((intertechnician OR inter-technician OR intratechnician OR intra-technician))) OR AB=((intertechnician OR inter-technician OR intratechnician OR intra-technician)) OR (TI=((interexaminer OR inter-examiner OR intraexaminer OR intra-examiner))) OR AB=((interexaminer OR inter-examiner OR intraexaminer OR intra-examiner))OR (TI=((interassay OR inter-assay OR intraassay OR intra-assay))) OR AB=((interassay OR inter-assay OR intraassay OR intra-assay)) OR (TI=((interindividual OR inter-individual OR intraindividual OR intra-individual))) OR AB=((interindividual OR inter-individual OR intraindividual OR intra-individual)) OR (TI=((interparticipant OR inter-participant OR intraparticipant OR intra-participant))) OR AB=((interparticipant OR inter-participant OR intraparticipant OR intra-participant)) OR (TI=(kappa)) OR AB=(kappa) OR (TI=(kappas)) OR AB=(kappas) OR TS=(repeatab*)OR TS=(((replicab* OR repeated) AND (measure OR measures OR findings OR result OR results OR test OR tests))) OR (TI=((generaliza* OR generalisa*))) OR AB=((generaliza* OR generalisa*)) OR (TI=(concordance)) OR AB=(concordance) OR (TI=((intraclass AND correlation*))) OR AB=((intraclass AND correlation*)) OR (TI=(discriminative)) OR AB=(discriminative) OR (TI=(“known group”)) OR AB=(“known group”) OR (TI=((“factor analysis” OR “factor analyses” OR “factor structure” OR “factor structures”))) OR AB=((“factor analysis” OR “factor analyses” OR “factor structure” OR “factor structures”)) OR (TI=(dimension*)) OR AB=(dimension*) OR (TI=(subscale*)) OR AB=(subscale*) OR (TI=((multitrait AND scaling AND (analysis OR analyses)))) OR AB=((multitrait AND scaling AND (analysis OR analyses))) OR (TI=(“item discriminant”)) OR AB=(“item discriminant”) OR (TI=(“interscale correlation*”)) OR AB=(“interscale correlation*”) OR (TI=((error OR errors))) OR AB=((error OR errors)) OR (TI=(“individual variability”)) OR AB=(“individual variability”) OR (TI=(“interval variability”)) OR AB=(“interval variability”) OR (TI=(“rate variability”)) OR AB=(“rate variability”) OR (TI=((variability AND (analysis OR values)))) OR AB=((variability AND (analysis OR values))) OR (TI=((uncertainty AND (measurement OR measuring)))) OR AB=((uncertainty AND (measurement OR measuring))) OR (TI=(“standard error of measurement”)) OR AB=(“standard error of measurement”) OR (TI=(sensitiv*)) OR AB=(sensitiv*) OR (TI=(responsive*)) OR AB=(responsive*) OR (TI=((limit AND detection))) OR AB=((limit AND detection)) OR (TI=(“minimal detectable concentration”)) OR AB=(“minimal detectable concentration”) OR (TI=(interpretab*)) OR AB=(interpretab*) OR (TI=(((minimal OR minimally OR clinical OR clinically) AND (important OR significant OR detectable) AND (change OR difference)))) OR AB=(((minimal OR minimally OR clinical OR clinically) AND (important OR significant OR detectable) AND (change OR difference))) OR (TI=((small* AND (real OR detectable) AND (change OR difference)))) OR AB=((small* AND (real OR detectable) AND (change OR difference))) OR (TI=(“meaningful change”)) OR AB=(“meaningful change”) OR (TI=(“ceiling effect”)) OR AB=(“ceiling effect”) OR (TI=(“floor effect”)) OR AB=(“floor effect”) OR (TI=(“item response model”)) OR AB=(“item response model”) OR (TI=(IRT)) OR AB=(IRT) OR (TI=(rasch)) OR AB=(rasch) OR (TI=(“differential item functioning”)) OR AB=(“differential item functioning”) OR (TI=(DIF)) OR AB=(DIF) OR (TI=(“computer adaptive testing”)) OR AB=(“computer adaptive testing”) OR (TI=(“item bank”)) OR AB=(“item bank”) OR (TI=(“cross-cultural equivalence”)) OR AB=(“cross-cultural equivalence”) |
