# Supplementary material for: Measurement properties of instruments used to measure health-related quality of life in pediatric and adults patients with inherited epidermolysis bullosa: A systematic review and meta-analysis protocol
Source: PLoS One. 2025 Sep 19;20(9):e0332844. doi: 10.1371/journal.pone.0332844 (PMC12448333; doi:10.1371/journal.pone.0332844)
Supplement: S4 Table — (DOCX) [file pone.0332844.s004.docx]

**S4 Table. Data extraction sheet**

|  | **Study characteristics** | | | | | | | **Characteristics of EB-specific HRQoL instruments** | | | | **Measurement properties** | | |
| --- | --- | --- | --- | --- | --- | --- | --- | --- | --- | --- | --- | --- | --- | --- |
| Author, year | Study (*design,*  *period*) | Objective(s) | Sample  characteristics (*size, sampling technique, sex, age, level of education, socioeconomic status…)* | Life stages (*pediatrics/ adults*) | EB subtype | Setting (*hospital-based, patient advocacy group…*) | Geographical area (*country, rural/ urban…)* | Instrument name | Mode of  administration | Dimensions | Items | Validity *(content, criterion and/or construct)* | Reliability *(test-retest, inter-rater, intra-rater and/or internal consistency)* | Other (*e.g. responsiveness, measurement error…)* |
|  |  |  |  |  |  |  |  |  |  |  |  |  |  |  |
|  |  |  |  |  |  |  |  |  |  |  |  |  |  |  |
|  |  |  |  |  |  |  |  |  |  |  |  |  |  |  |
|  |  |  |  |  |  |  |  |  |  |  |  |  |  |  |
|  |  |  |  |  |  |  |  |  |  |  |  |  |  |  |
|  |  |  |  |  |  |  |  |  |  |  |  |  |  |  |
|  |  |  |  |  |  |  |  |  |  |  |  |  |  |  |
|  |  |  |  |  |  |  |  |  |  |  |  |  |  |  |
|  |  |  |  |  |  |  |  |  |  |  |  |  |  |  |
|  |  |  |  |  |  |  |  |  |  |  |  |  |  |  |
|  |  |  |  |  |  |  |  |  |  |  |  |  |  |  |
|  |  |  |  |  |  |  |  |  |  |  |  |  |  |  |
|  |  |  |  |  |  |  |  |  |  |  |  |  |  |  |
|  |  |  |  |  |  |  |  |  |  |  |  |  |  |  |

EB: Epidermolysis Bullosa; HRQoL: Health-related quality of life.
